# Phimosis

**Published:** 1897-04-24

**Authors:** W. McAdam Eccles

**Affiliations:** Assistant Surgeon West London Hospital, City of London Truss Society, &c.


### PHIMOSIS.

By W. McAdam Eccles, M.S.(Lond.), F.R.C.S.(Eng.), Assistant Surgeon West London Hospital, City of London Truss Society, &c.

Phimosis is, perhaps, one of the commonest of congenital malformations; in fact, so frequent is it as to be considered by many as hardly of sufficient importance to be ranked as a defect. But conditions slight in themselves may lead to serious consequences, and phimosis is one of such.

An elongated prepuce is a disadvantage, but when associated with a narrow orifice its untoward results may be very marked. This is a form of phimosis which is being constantly met with.

On the other hand there may be seen a prepuce which is, perhaps, rather shorter than usual, and with no contraction of its opening, but with adhesion of the skin and the glans penis. This is a natural condition present at birth, and one which generally disappears of itself. Strictly speaking, it should not come under the category of phimosis. A director or a probe will, in the majority of cases, suffice to break down the attachment if it become necessary to do so, but care must be afterwards taken not to allow fresh union to occur. This can be prevented by the application for a few days of some simple ointment spread on a strip of soft aseptic gauze. Most frequently, however, phimosis occurs as a result of a narrow preputial orifice without any marked lengthening of the foreskin, or adhesion to the glans. So constricted may the opening be in some of the cases, that it is with great difficulty that it is discovered, or a probe passed through it.

Frequent and difficult micturition as a rule attends such cases, the infant commonly exhibiting signs of distress during the act. Retention of urine may occur; or distension of the bladder, with overflow, leading to what is often termed incontinence. Enuresis and occasionally priapism follow in the train. The prepuce may be distended or ballooned when urine is voided.

It is said that inguinal hernia often arises in consequence of the straining efforts of the child. This may be true in some infants who have a patent processus vaginalis, but it certainly does not very often occur when these peritoneal prolongations have become obliterated; and, moreover, it must be freely admitted that a very large number of cases of aggravated phimosis are not the subjects of hernia. Thus, although the condition may play a part in the production of hernia, it is probably but rarely the sole factor.

Prolapse of the rectum, a common ailment in children, is also in some instances induced by pre-existing phimosis. The baneful effects of the obstruction to the outflow from the bladder upon that organ itself are not slow in showing themselves. Hypertrophy, with marked fasiculation of the muscular tissue, is common. Sacculation may even in some cases occur. Inflammation of the bladder may be induced, particularly in neglected cases. Further, the backward pressure may act on the ureters, bringing about their dilatation, and, later on, that of the pelves of the kidneys themselves. Sometimes marked double hydronephrosis may ensue.

Children the subjects of phimosis are often very irritable, and there is a constant tendency for a habit of manipulating the parts to be contracted, thus producing the most undesirable consequences. Loss of appetite, and, therefore, loss of flesh, not infrequently occurs, and even the boy's proper development of mind and body may be impaired. Convulsions, epilepsy, and other nervous phenomena at times show themselves. Various affections of the prepuce itself and surrounding parts may result; thus preputial calculi, thickening of the prepuce from repeated balanitis, paraphimosis, rupture of the frænum, and, in after life, epithelioma are all to be seen as an outcome of phimosis. Imperfect coitus is not uncommon. From the position assumed by the boy in micturition, namely, with the legs much abducted, some deformity of the feet, and simulated or actual morbus coxæ may rarely follow.

Seeing then that phimosis, although apparently so trivial a matter, is so common and not unlikely to lead to serious results, its treatment becomes a matter of great importance. There are three methods in common use. Firstly, to dilate the preputial orifice, separate the adhesions, and leave the part further untouched. Secondly, to slit the prepuce along the dorsum in the middle line, and break down the adhesions. Thirdly, to circumcise.

With regard to the first procedure, there is no doubt that dilatation will temporarily improve matters, but there is a great tendency towards recontraction, and, moreover, the separated adhesions nearly always re-form. Even if a degree of permanent dilation remains, the patient still has a prepuce which will probably give him trouble from balanitis, paraphimosis, retention of secretions, &c.

The second method has nothing to recommend it, in fact everything to condemn it, for it does not radically remove the source of the trouble, and adds to it the very unsightly condition of a split prepuce.

Circumcision is, therefore, probably by far the best means of cure in all cases of phimosis, and when carried out in an infant and in a proper manner it gives every satisfaction.

The operation ensures the subsequent free passage of urine, the cessation of all irritation from underlying smegma, and lessens the tendency to the formation of undesirable habits. It is especially to be undertaken in long or very tight prepuces, where adhesions have recurred, and where much balanitis persists. Whether carried out on the infant or the adult, the principles of the procedure are much the same. Some of the minor details differ, and these will be mentioned in passing.

A general anæsthetic is advisable—chloroform for the infant, ether for the adult—for cocaine and other local anæsthetics are somewhat uncertain, and the area to be dealt with is a very sensitive one. A thorough washing of the parts with soap and water is most essential, and this should not be a mere wipe over, but a very deliberate cleansing. When the soap has been rinsed off, a solution of 1-2,000 perchloride of mercury is rubbed over the whole region, and a square of some little thickness, and about six to eight inches on each side, should be cut from sal alembroth gauze, or lint. This is wrung out of the perchloride solution used above, and a small hole, just large enough to pass the penis through, is to be made in its centre. Thus used, the square acts in two ways—firstly, as an antiseptic area within which the operation is performed, and, secondly, by compressing the root of the penis to a certain extent, it plays the part of a tourniquet, and also absorb any blood which may escape.

There are two pitfalls which are to be avoided in performing this little operation. Too little or too much may be removed. If the former, the patient is not much better off than if nothing had been undertaken; if the latter, deformity may result on the contraction of the scar.

What, then, is the best way of securing the removal of the proper amount of tissue? In the child, it is accomplished by noting or, better, marking with an aniline pencil the line of the corona of the glans, and then taking a pair of long straight-bladed forceps and adjusting them to the skin at this level. While the blades are being shut, it will be found that the skin slips forwards and the glans backwards, till at last the forceps retain nothing but the prepuce. The part beyond the forceps is now put off the stretch by drawing it forwards, and cut off with a sharp knife or pair of scissors. In this way all danger of wounding the glans is avoided. As soon as the forceps are removed the skin retracts to the posterior part of the corona, leaving the glans covered only by a layer of mucous membrane. This is next slit up with a pair of scissors along the median line back to the corona. Some difficulty may now be experienced in separating the mucous membrane from the glans, to which it may be very firmly adherent. Peeling it back with the thumbs or detaching it with a director is the best means to be employed. Care must be taken while doing this not to injure the tissue of the glans. The little flaps thus formed are to be trimmed off on either side, but the frænum, if possible, should not be cut through. Any retained smegma must now be carefully washed away with the perchloride solution. No vessels usually need tying. In an adult it is perhaps preferable to pass a director between the glans and the prepuce, and slit the latter along the dorsum back to the corona. The director must on no account be passed into the urethra by accident. Each side may now be rounded off along the line of the corona. One or two vessels may need to be dealt with. Both in the infant and in the adult I am in the habit of inserting a sufficient number of silk or catgut sutures so as to ensure that all the raw surface is covered in, for in this way healing by primary union may be achieved. If no sutures are used there is a strong likelihood of the surfaces being separated and healing being thus delayed.

As a dressing nothing answers better than a strip of dry gauze, which controls oozing, remains firmly in position, and keeps the cut edges accurately in contact.

The dressing may be frequently changed—in a child twice a day or even oftener—by soaking it off in hot water. An adult should not be deprived of the luxury of a bath longer than two days, and may leave his bed for good on the third or fourth day after the operation.

By the fifth or sixth day all parts should be satisfactorily united. If attention to asepsis is neglected, suppuration, cellulitis, sloughing, and erysipelas may result. If too little skin be removed, especially on the lower aspect, there is much liability for a thickened, indurated mass to remain. This result should be avoided by carefully following out the details which have been given. I have known it necessitate a second operation for its cure.

Finally, in an adult some discomfort will arise for the first few weeks owing to the tenderness of the exposed glans, a fact of which a patient should always be warned, but with a little care no harm will come of it.

